# Dangerous Cosmetics

**Published:** 1861-09

**Authors:** 


DANGEROUS COSMETICS.—At a recent sitting of the French Academy of Medicine, Dr. Réveil read a paper "On the Necessity of Preventing Perfumers from Selling Poisonous or Dangerous Articles," which should be exclusively left to the responsibility of regular chemists, and not sold without a physician's prescription. "To show the danger there is in allowing the unchecked sale of certain compounds," he said, "I need but state that arsenic, the acid nitrate of mercury, tartar emetic, cantharides, colchicum, and potassa caustica form part of their ingredients. The kind of soap called lettuce soap, which is sold with the announcement that it has been acknowledged by the Academy, does not contain the slightest trace of lettuce. This and other soaps are all colored green by the sesquioxide of chromium, or of a rose color by the bisulphuret of mercury, known as vermilion. Some which are cheaper contain 30 per cent. of insoluble matter, such as lime or plaster, while others contain animal nitrogenous matter which, having escaped the process of saponification, emits a bad smell when its solution is left exposed to the air. The various toilet vinegars are so far noxious that, being applied to the skin still impregnated with soap and water, they give rise to a decomposition, in consequence of which the fatty acids of soaps, being insoluble in water, are not removed by washing, become rancid, and cause a chronic inflammation of the skin. The preparations employed for hair-dye under the pompous names of 'African water,' 'Florida water,' etc., all contain nitrate of silver, sulphur, oxide and acetate of lead, sulphate of copper, and other noxious substances. All cosmetics for removing hair or freckles are dangerous; the *lait antéphélique*, for instance, contains corrosive sublimate and oxide of lead. Were a chemist to deliver such remedy to a customer without a regular prescription, he would be liable to a fine of 6000f." Dr. Réveil concluded by expressing his regret that certain physicians should so far forget their own dignity as to lend the support of their names to such noxious inventions.—*Galignani's Messenger.*

